# A Moderated Mediation Model of Academic Supervisor Developmental Feedback and Postgraduate Student Creativity: Evidence from China

**DOI:** 10.3390/bs12120484

**Published:** 2022-11-29

**Authors:** Weilin Su, Qian Qi, Shuai Yuan

**Affiliations:** 1 School of Literature, Capital Normal University, Beijing 100089, China; 2 School of Artificial Intelligence, Beijing University of Posts and Telecommunications, Beijing 100876, China; 3 School of Management Engineering and Business, Hebei University of Engineering, Handan 056009, China

**Keywords:** academic supervisor developmental feedback, creative self-efficacy, intrinsic motivation, postgraduate student creativity

## Abstract

Academic supervisors plays a significant role in the cultivation of postgraduate students, but little is known about how academic supervisor feedback affects their creativity. This study hypothesizes and tests a moderated mediation model to explore how and when academic supervisor developmental feedback (ASDF) affects postgraduate student creativity (PSC), including the mediating effect of intrinsic motivation and the moderating effect of creative self-efficacy. After collecting three-wave time-lagged data from 374 postgraduate students and their academic supervisors, SPSS and Amos software were used to test the research hypotheses and the whole model. The results show that ASDF is positively related to intrinsic motivation and PSC. Intrinsic motivation not only has a positive effect on PSC, but it also plays a mediating role in the relationship between ASDF and PSC. Creative self-efficacy plays a moderating role in the relationships between ASDF, intrinsic motivation, and PSC, that is, ASDF can cause postgraduate students with high creative self-efficacy to develop higher levels of intrinsic motivation than those with low creative self-efficacy, which ultimately leads to more PSC. These findings not only enrich the literature on feedback, motivation, and creativity research in the field of education, but also provide some suggestions for promoting PSC from the perspective of universities, academic supervisors, and postgraduate students.

## 1. Introduction

Postgraduate education is the main way to cultivate innovative talents [1], and postgraduate students are a significant reserve force for building an innovative country [2]. As the main institutions of postgraduate training, colleges and universities have been shouldering the mission of fostering creators and generating creativity [3,4], and academic supervisors of postgraduates are exactly the front-line leaders to undertake this mission [5]. Much of the prior research has confirmed that developing postgraduate student creativity (PSC) is quite complicated [6,7], and the leadership behavior or style of the direct academic supervisor is one of the most important factors affecting the creativity of postgraduate students. For example, Meng and Zhao propose that academic supervisors’ empowering, considerate, motivating, and developing behaviors exert a positive influence on PSC [8]. Shang and colleagues verify that authentic leadership by academic supervisors is positively correlated with PSC [9]. Nevertheless, such research usually presents the characteristics of fragmentation and functionalization and tends to focus on the active output of academic supervisors, paying insufficient attention to the interactions between academic supervisors and postgraduate students [10].

In fact, academic supervisor feedback is essential in every academic institution [11]. When conducting academic research activities, postgraduate students prefer to seek feedback from their academic supervisors rather than from their classmates and friends. In other words, compared with feedback from other sources, feedback from academic supervisors has the most significant impact on postgraduate students [12]. Academic supervisor developmental feedback (ASDF) refers to the extent to which academic supervisors provide postgraduate students with helpful and useful information that enables postgraduate students to learn, develop, and make improvements [13]. It emphasizes the two-way interaction between academic supervisors and postgraduate students [14]. When postgraduate students perceive ASDF, they usually believe that their academic supervisor cares for, trusts, and supports them, and they have the idea of “returning the favor”, which ultimately motivates them to participate in academic research and improve their creativity. Additionally, as the individuals who have the closest contact with postgraduate students [15], academic tutors can create a relaxed and pleasant atmosphere through ASDF, thus promoting the creativity of postgraduate student. Therefore, this study expects that ASDF has a positive effect on PSC.

Many previous studies have also pointed out that PSC is the results of the interaction between extrinsic situational factors and individual internal factors [6,16]. Only when the effect of the academic supervisor is transformed into the internal cause of a certain level of development in a postgraduate student can it really promote PSC [17]. Hence, individual factors affecting postgraduate students may be the mediating factors of the relationship between ASDF and PSC. Additionally, intrinsic motivation, as the internal driving force for individuals to participate in creative activities [18], has been considered by numerous scholars an important mediating mechanism which explains the influence of academic supervisor leadership or behavior on PSC [8,19]. In addition, intrinsic motivation is the result of the combination of external situational factors and individual characteristics [20,21]. Creative self-efficacy refers to the belief in one’s own ability to achieve creative results [22,23], and it plays a positive role in the formation of individual intrinsic motivation [24]. Therefore, this study concludes that the creative self-efficacy of postgraduate students may exert a moderating role in the relationships between ASDF and intrinsic motivation and affect PSC.

**Figure 1:**
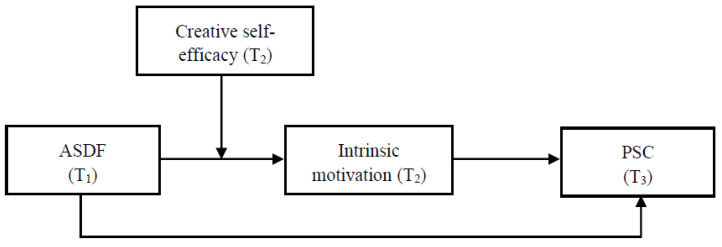
The proposed research model.

To sum up, this study aims to address the questions of “how” and “when” ASDF affects PSC. Using a multi-time sample of postgraduate student–academic supervisor dyads to examine the moderated mediation theoretical model (see Figure 1), this study builds on the literature in several ways. First, this study contributes to the literature on feedback research by focusing on the direct effects of ASDF on intrinsic motivation and PSC. Additionally, through introducing intrinsic motivation as a mediator, this study opens the black box of how ASDF affects PSC and expands the research on individual motivation. In addition, this study broadens the research on the boundary condition of ASDF–intrinsic motivation–PSC by exploring the moderating role of creative self-efficacy.

## 2. Theoretical Framework and Hypotheses

### 2.1. ASDF

Developmental feedback refers to the extent to which supervisors provide useful information to subordinates to enable the subordinates to learn, develop and improve in their future work [13]. In essence, ASDF is the specific application of the concept of supervisor development feedback in the field of education, especially in the academic supervisor–postgraduate student relationship. Consistent with developmental feedback, ASDF also has three distinctive characteristics: first, it comes from academic supervisors who are directly responsible for postgraduate students, and they usually change the form and content of feedback to suit the characteristics of individual postgraduate students. Second, the specific content of ASDF is developable and valuable, and thus can effectively help postgraduate students to learn, develop, and improve. Third, ASDF belongs to the information feedback mode and does not involve a mandatory completion time [14,25]. Many previous studies have confirmed the positive influence of developmental feedback from direct supervisors on individuals’ intrinsic motivation [26,27,28] and creativity [13,29,30]. Therefore, it is reasonable to conclude that ASDF has a positive impact on the intrinsic motivation of postgraduate students and PSC.

### 2.2. PSC

Research on individual creativity began in the psychology field in the 1950s, and then spread to the fields of education and management [31]. There are many different understandings of the concept of creativity in theoretical circles, but its essential connotation is the same. Most studies define it as “novel and practical ideas or concepts that individuals build on existing products, processes, and services in a specific organization” [32] and measure it by evaluating individuals who have advanced knowledge in a field of interest [33]. For PSC, Amabile and colleagues proposed that its basic components include passion, cognition, motivation, belief acceptance, belief pursuit, knowledge transformation, and application [34]. Wang and her collaborators define it as the willingness of postgraduate students to seek knowledge and explore new things, as well as their ability to creatively propose new ideas, apply new methods, create new things, and solve new problems by integrating knowledge and experience [2]. In line with these studies, this study therefore believes that PSC refers to the ability of postgraduates to make full use of their knowledge and experience, to form new thinking, new methods, and new skills in the process of exploring the unknown, and to create new things by using them under the guidance of their academic supervisors.

### 2.3. ASDF and PSC

As mentioned above, ASDF is essentially informational feedback [11,13] which can provide useful information for postgraduate students. Through the information conveyed by ASDF, postgraduate students gain relevant knowledge and creative skills in their professional fields. These new perspectives and this new knowledge from their academic supervisors help postgraduate students “think out of the Box” and inspire them to produce creative ideas [35,36]. Additionally, ASDF is long-term oriented [37]. Under such guidance, postgraduate students are not limited to short-term gains and losses, but are willing to invest time and energy to learn innovative skills, and then actively generate creative ideas to solve problems and improve themselves. In addition, when postgraduate students perceive ASDF, they usually generate a sense of living and studying in a relaxed and free atmosphere [38] and are more daring to break with routine and try new methods without being afraid of the risks associated with innovation [14,29], and are thus more likely to show high creativity. Therefore, this study posits that:

**H1:**  *ASDF is positively related to PSC*.

### 2.4. Mediating Effect of Intrinsic Motivation

Motivation is the deep-seated reason for individuals to act and make efforts to achieve certain goals [39], and it can be divided into extrinsic motivation and intrinsic motivation [40]. Extrinsic motivation emphasizes the desire to gain external approval (such as a bonus) or to avoid external threats (such as scolding). Intrinsic motivation focuses on the willingness of individuals to participate in certain activities for their own interest and pleasure [19,41]. Many previous studies have proved that individuals’ perception of competence and autonomy are the two key components of their intrinsic motivation [42,43], and these are likely to be influenced by leadership style or supervisor behavior [8,13]. Based on the logic of these studies, this study predicts that when postgraduate students perceive the learning, development, and improvement information provided by ASDF, they generally experience a sense of competence and autonomy being satisfied to a large extent [19,27], and their intrinsic motivation is also continuously enhanced during this process.

Additionally, as an important individual factor affecting how postgraduate students actively study, work, or engage in research [21], intrinsic motivation has an important impact on their creative ideas, thinking, behavior, and performance [2,6,44]. Specifically, driven by intrinsic motivation, postgraduate students usually show strong interest, enthusiasm, and confidence in their daily life [43], and this promotes their effort and investment in creative problem-solving activities and ultimately promotes their creativity [17,31]. In addition, prior studies have confirmed that the intrinsic motivation of postgraduate students is easily affected by external environmental factors [45]. It has been an important mediating variable for conveying the influence of academic supervisors’ leadership behavior or style on PSC (e.g., [6,8,19]). Therefore, this study infers that ASDF does not manipulate and control postgraduate students, but stimulates the progress and growth of postgraduate students through the transmission of innovation-related developmental information, further stimulates the intrinsic motivation of postgraduate students to generate creative ideas and participate in creative activities, and finally promotes PSC. According to the above arguments, this study posits that:

**H2:**  *ASDF is positively related to the intrinsic motivation of postgraduate students*.

**H3:**  *The intrinsic motivation of postgraduate students is positively related to PSC*.

**H4:**  *The intrinsic motivation of postgraduate students mediates the relationship between ASDF and PSC*.

### 2.5. Moderating Effect of Creative Self-Efficacy

Creative self-efficacy, as an extension of general self-efficacy in the creativity domain, refers to the belief in one’s ability to achieve creative results [46]. It has always been assumed to be an indispensable attribute in individual creative work and plays an important role in promoting individual creativity [45]. Growing empirical evidence has verified that creative self-efficacy can significantly motivate individuals to generate and execute creative ideas [47,48] and to actively cope with various difficulties and uncertainties in the creative process [19,49,50]. That is, individuals with high creative self-efficacy are more confident in their ability to achieve positive outcomes, are more likely to perceive supportive and positive information in the external environment [14], and thus are more motivated to participate in creative activities and ultimately exhibit a higher level of creativity. This study therefore predicts that the creative self-efficacy of postgraduate students may play an important moderating role in the relationship between ASDF and intrinsic motivation, and also affect PSC.

Specifically, postgraduate students with high creative self-efficacy tend to be more willing to participate in creative work activities spontaneously because they have high confidence in their ability to complete creative tasks [51]. When they perceive ASFG, they usually generate more intrinsic motivation to overcome difficulties and engage in more creative activities [13,45], and their creativity is naturally enhanced in this process [52]. In contrast, postgraduate students with low creative self-efficacy tend to doubt their ability to complete creative tasks or engage in creative activities [53]. In the face of ASDF, due to their lack of belief in the success of innovation [54], they usually choose to ignore creative ideas or thinking, and even suppress their intrinsic motivation to participate in creative activities to avoid the possibility of failure, so their creativity is unlikely to be enhanced. Hence, the creative self-efficacy of postgraduate students not only moderates the influence of ASDF on intrinsic motivation, but also moderates the mediating effect of intrinsic motivation in the relationship between ASDF and PSC. Taken together, this study posits that:

**H5:**  *The creative self-efficacy of postgraduate students moderates the relationship between ASDF and their intrinsic motivation. In other words, the relationship is stronger for postgraduate students with higher creative self-efficacy than for those with lower creative self-efficacy*.

**H6:**  *The creative self-efficacy of postgraduate students moderates the indirect effect of ASDF on PSC via intrinsic motivation. In other words, this indirect relationship is stronger for students with higher creative self-efficacy than for those with lower creative self-efficacy*.

## 3. Methods

### 3.1. Participants and Sampling Procedure

To test the research hypotheses and the whole theoretical model, this study conducted an empirical survey among academic supervisors and postgraduate students in a leading research university in Beijing, China. This university has an enrollment of more than 28,000 students, including 12,167 undergraduates, 7329 master’s students and 1360 doctoral students. It has formed a comprehensive and multi-level educational structure and system from junior college to undergraduate, master’s, doctoral, and post-doctoral, and from full-time to adult education and international education. Furthermore, as many previous studies have confirmed, urban schools have the greatest supply of teachers, higher teaching quality, and diverse educational resources compared with those in rural areas [9]. This study therefore considers the collected data sample to be representative as it samples high-level postgraduate students and their academic supervisors from a leading research university in one of the core cities in Mainland China. 

Specifically, with the help of educational administrators from the graduate school, this study first obtained a list of all the current postgraduate students and their academic supervisors at this university. Then, 500 postgraduate students and their academic supervisors were randomly selected to participate in this empirical survey. All survey scales and their links were sent to participating postgraduate students and their academic supervisors via Enterprise WeChat, which has been widely used for online education at this university during the COVID-19 pandemic. Furthermore, in order to enhance the reliability and reduce common method bias, this study collected data from multiple sources (based on the scores of postgraduate students and their academic supervisors on the questionnaire items) at three different times. Consistent with previous studies [9,54,55], the sample data were collected every two weeks. At time 1, the postgraduate students were asked to report their perceptions of developmental feedback from their academic supervisors, creative self-efficacy, and demographic information. At time 2, the postgraduate students were asked to fill in a questionnaire containing items relating to intrinsic motivation. At time 3, the academic supervisors (assistant professors, associate professors, and professors) were invited to evaluate the creativity of their own postgraduate students. 

A total of 374 matched sample data were obtained in this study, excluding invalid questionnaires that could not be matched, had some missing answers, or had obvious regularity of choices, and so on, yielding a response rate of 74.8%. Among the final valid sample, 169 (45.19%) were males and 205 (54.81%) were females. Regarding the grade, the distribution was relatively even: 127 (33.96%) were first-year graduate students, 139 (37.16%) were second-year graduate students, and 108 (28.88%) were third-year graduate students. Regarding the major, 39 (10.43%) were majoring in philosophy, 64 (17.12%) were majoring in math, 71 (18.98%) were majoring in history, 74 (19.78%) were majoring in education, 69 (18.45%) were majoring in literature, 43 (11.49%) were majoring in management, and 14 (3.75%) were majoring in other subjects.

### 3.2. Measures

The main variables involved in this study, ASDF, creative self-efficacy, intrinsic motivation, and PSC, were measured by mature scales in English. Hence, two bilingual researchers (Chinese and English) were invited to translate all measurement items into Chinese and then back into English following standard translation and backtranslation procedures. This study also asked two translators to comment on any vaguely worded items, but neither suggested any notable changes. All the responses were formatted as 5-point Likert scales ranging from 1 = “strongly disagree” to 5 = “strongly agree”.

#### 3.2.1. ASDF

A three-item scale developed by Zhou was used to measure the postgraduate students’ perception of supervisor developmental feedback at Time 1 [13]. One sample item was “My academic supervisor often provides me with feedback that helps me grow and develop”. The Cronbach’s alpha for this measure was 0.736.

#### 3.2.2. Intrinsic Motivation

The four-item scale developed by Amabile et al. was adopted to test the intrinsic motivation of the postgraduate students at Time 2 [18]. Sample items included “It’s important for me to have an outlet to express myself” and “I am much happier when I can set goals for myself”. The Cronbach’s alpha for this measure was 0.826.

#### 3.2.3. Creative Self-Efficacy

The three-item scale developed by Tierney and Farmer was used to test the creative self-efficacy of the postgraduate students at Time 2 [46]. Sample items included “I’m pretty good at generating new ideas” and “I believe I can solve problems creatively”. The Cronbach’s alpha for this measure was 0.825.

#### 3.2.4. PSC

The four-item scale developed by Farmer et al. was applied to evaluate PSC as perceived by academic supervisors at Time 3 [56]. Sample items included “This student often tries new ideas or methods in daily life” and “This student is a good example of creativity”. The Cronbach’s alpha for this measure was 0.836.

#### 3.2.5. Control Variables

On the basis of previous studies, the gender, grade, and major of postgraduate students were selected as the control variables in this study [2,8,13,37,44]. Gender was a binary variable coded as 0 = Male and 1 = Female; the grade of the participants was divided into three levels (1 = First year, 2 = Second year, and 3 = Third year). The major of the participants was divided into seven types (1 = Philosophy, 2 = Math, 3 = History, 4 = Education, 5 = Literature, 6 = Management, and 7 = Others).

### 3.3. Data Analysis

All statistical analyses in this study were performed using SPSS 21.0 and Amos 22.0 software. Specifically, this study first conducted confirmatory factor analyses (CFAs) to check the discriminant validity of the theoretical model using Amos 22.0. It is usually measured using the ratio of chi-Square/degree of freedom (χ^2^/df), the comparative fit index (CFI), the goodness of fit index (GFI), the root mean square error of approximation (RMSEA), and the standardized root mean square residua (SRMR) [57]. Additionally, descriptive statistics, correlation, and hierarchical regression were used to preliminarily test the relationships between ASDF, creative self-efficacy, intrinsic motivation, and PSC using SPSS 21.0 (IBM, Beijing, China). Finally, the current study used bootstrap methods via the PROCESS program developed by Hayes with Model 7 to further verify the whole moderated mediation model [58].

## 4. Results

### 4.1. Confirmatory Factor Analyses

To check whether ASDF, intrinsic motivation, creative self-efficacy, and PSC could be mutually discriminated, this study used Amos 22.0 (IBM, Beijing, China) to conduct CFAs (see Table 1). The results show that the hypothesized four-factor model fitted well with the data (χ^2^/*df* = 1.067 < 3, CFI = 0.998 > 0.9, GFI = 0.972 > 0.9, RMSEA = 0.013 < 0.08, SRMR = 0.044 < 0.08), and significantly better that the three-factor model (χ^2^/*df* = 4.159, CFI = 0.884, GFI = 0.868, RMSEA = 0.092, SRMR = 0.088), the two-factor model (χ^2^/*df* = 5.880, CFI = 0.824, GFI = 0.824, RMSEA = 0.114, SRMR = 0.113), and the single-factor model (χ^2^/*df* = 11.987, CFI = 0.599, GFI = 0.677, RMSEA = 0.172, SRMR = 0.188). Hence, the discriminant validity of this study is good and suitable for subsequent hypotheses testing.

### 4.2. Descriptive Statistics and Correlation Analyses

Table 2 reports the results of the descriptive statistics and correlation analyses, including the means, standard deviations, and intercorrelations between the studied variables. It shows that ASDF is positively related to intrinsic motivation (*r* = 0.390, *p* < 0.01) and PSC (*r* = 0.337, *p* < 0.01). Intrinsic motivation is also positively related to PSC (*r* = 0.289, *p* < 0.01). Hence, these results provide preliminary evidence for the six proposed hypotheses. 

### 4.3. Hypotheses Testing

#### 4.3.1. Direct Effects Testing 

Table 3 reports the hierarchical regression results of the main study variables. As Model 7 shows, the influence coefficient of ASDF on PSC is significantly positive (*β* = 0.218, *p* < 0.001), indicating that ASDF has a positive direct effect on PSC, thereby supporting Hypothesis 1.

In Model 2, the influence coefficient of ASDF on intrinsic motivation is also significantly positive (*β* = 0.349, *p* < 0.001), indicating that ASDF has a positive direct effect on the intrinsic motivation of postgraduate students. Additionally, the influence coefficient of intrinsic motivation on PSC in Model 5 is significantly positive (*β* = 0.208, *p* < 0.001), indicating that the intrinsic motivation of postgraduate students exerts a positive direct effect on PSC. Hence, both Hypothesis 2 and Hypothesis 3 are supported.

#### 4.3.2. Mediating Effect Testing

In order to check whether intrinsic motivation served as a mediator for the relationship between ASDF and PSC, this study followed Preacher and Hayes’ procedures for justifying a mediating effect [59]. From the perspective of this research, firstly, ASD should be significantly related to intrinsic motivation. Secondly, after controlling the direct effect of ASDF on PSC, the relationship between intrinsic motivation and PSC should be significant, and the indirect effect of ASDF on PSC must be significant as well. The results of Model 2 in Table 3 satisfy the first criterion. In addition, after adding intrinsic motivation to the regression model, the results of Model 8 in Table 3 reveal that intrinsic motivation positively predicts PSC (*β* = 0.167, *p* < 0.01) and that ASDF still has a significant effect on PSC (*β* = 0.161, *p* < 0.01). Therefore, the intrinsic motivation of postgraduate students plays a partial mediating role in the relationship between ASDF and PSC, supporting Hypothesis 4.

#### 4.3.3. Moderating Effect Testing

**Figure 2:**
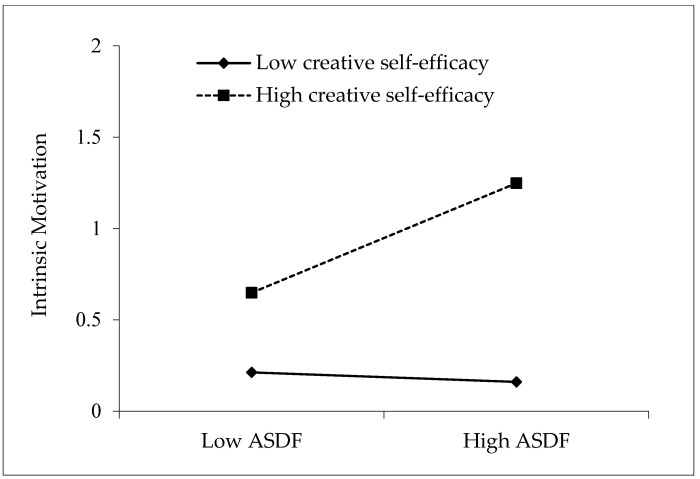
Moderating effect of creative self-efficacy in the relationship between ASDF and intrinsic motivation.

In order to test whether creative self-efficacy served as a moderator for the relationship between ASDF and intrinsic motivation, this study adopted procedures for justifying a moderating effect. The results of Model 3 in Table 3 show that the interaction term of ASDF and creative self-efficacy is significant in predicting intrinsic motivation (*β* = 0.381, *p* < 0.001), which means that creative self-efficacy positively moderates the effect of ASDF on intrinsic motivation. To demonstrate this moderating effect more intuitively, the mean value of creative self-efficacy was further added and subtracted by one standard, and the sample data were divided into two groups: high creative self-efficacy and low creative self-efficacy, as presented in Figure 2. As is shown, ASDF has a greater positive effect on the intrinsic motivation of postgraduate students with high creative self-efficacy than on those with low creative self-efficacy. Thus, Hypothesis 5 is supported.

#### 4.3.4. Moderated Mediation Effect Testing

In order to check the overall theoretical model, this study employs bootstrap methods to test the moderating role of creative self-efficacy for the indirect effect of ASDF on PSC via intrinsic motivation [60]. The analytical results are presented in Table 4. As is shown, the effect of creative self-efficacy on the moderated mediation effect is significant (*β* = 0.025, SE = 0.019, 95% CI = 0.007–0.054). Additionally, the mediating effect of intrinsic motivation on the ASDF–PSC relationship is stronger when the deficit correction is high (*β* = 0.051, CI = 0.018, 0.106), but not when the deficit correction is low (*β* = 0.002, CI = −0.027, 0.313). In other words, creative self-efficacy positively moderates the mediating effect in the relationship between ASDF and PSC. Taken together, Hypothesis 6 and the whole moderated mediation model are fully supported.

## 5. Discussion

The main goal of this study was to investigate how and when ASDF affects PSC. Specifically, the current study designed and validated a moderated mediation model to discuss the mediating effect of intrinsic motivation and the moderating effect of creative self-efficacy in the relationship between ASDF and PSC. The empirical results indicate that ASDF has a positive influence on the intrinsic motivation of postgraduate students and PSC. Intrinsic motivation not only exerts a positive effect on PSC, but also mediates the influence of ASDF on PSC. Creative self-efficacy positively moderates the effect of ASDF on intrinsic motivation and also affects PSC, which means that for postgraduate students with high creative self-efficacy, ASDF stimulates their intrinsic motivation more obviously, and their creativity is higher than those with low creative self-efficacy. Taken together, these conclusions enrich the current literature on feedback, motivation, and creativity by examining the mediating effect of intrinsic motivation and the moderating effect of creative self-efficacy in the association between ASDF and PSC.

### 5.1. Theoretical Implications

This study provides theoretical insights by further demonstrating the importance of creative self-efficacy and intrinsic motivation in the influence processes of ASDF on PSC. Hence, the findings of the current study make contributions by proposing a moderated mediation model to identify key factors that help promote PSC in higher education from the perspective of feedback, motivation, and self-efficacy.

First, this study verified that ASDF could enhance PSC. In other words, the more developmental feedback postgraduate students receive from their academic supervisors, the more creativity they show in their daily lives. Previous studies have verified a positive relationship between developmental feedback and subordinate creativity in the context of organizational management [13,29,46]. However, in terms of their specific application of the relationship between supervisor and subordinate in the field of education [61], as far as we know, the existing studies on the impact of ASDF on PAS are not sufficient. Therefore, this conclusion not only responds to the call of prior scholars to strengthen the research on the influence of academic supervisor behavior or leadership style on PSC in the context of Chinese higher education, but it also further deepens the understanding of ASDF, thus enriching the existing literature on developmental feedback.

Second, this study introduced intrinsic motivation as a mediating variable to further explain the internal influence process of ASDF on PSC. The empirical test results indicate that when postgraduate students perceive developmental feedback from their academic supervisors, their competence and autonomy at work are effectively improved, which further stimulates their intrinsic motivation to take an active part in creative activities and show a higher level of creativity. Consistent with previous studies in the Chinese context [8,19], this conclusion provides new supporting evidence that the intrinsic motivation of postgraduate students has been an important mediating factor in the influence of academic supervisor leadership style or behavior affecting PSC. In addition, the mediating role of intrinsic motivation also provides an explanatory framework for subsequent research on the relationship between academic supervisor–student creativity, which further enriches and improves the related research on motivation theory in the field of education [2,40,43].

Third, this study confirmed that creative self-efficacy, as individuals’ belief in their ability to achieve creative results [23,24,46,51], plays a moderating role in the relationship between ASDF, intrinsic motivation, and PSC. Specifically, the creative self-efficacy of postgraduate students can effectively enhance the effect of ASDF on their intrinsic motivation and creativity. That is, the stronger their creative self-efficacy, the more significant the positive effect of ASDF on their intrinsic motivation, which can lead to more PSC. Together with previous studies [7,19,44], this conclusion broadens the research on the boundary condition of ASDF–intrinsic motivation–PSC and provides a new basis for educational scholars to understand the conditions under which academic supervisors affect the motivation and creativity of postgraduate students.

### 5.2. Managerial Implications

In the academic careers of postgraduate students, their closest relationships are with their academic supervisors [12,62]. The leadership style or behavior of an academic supervisor has a significant impact on PSC [51]. Therefore, based on previous studies and research findings, this study also provides some suggestions for improving the creativity of postgraduate students.

First, considering the positive effect of ASDF on intrinsic motivation and PSC, academic supervisors should be fully aware of the importance of giving developmental feedback to postgraduate students. In the process of daily communication with postgraduates, academic supervisors should pay attention to the development of feedback content on the one hand, and take the initiative to provide postgraduates with detailed, targeted, and high-quality feedback that can help them learn, grow, and improve in the future. On the other hand, academic supervisors should guide postgraduates to identify valuable research problems, help them overcome the difficulties involved in study, life, and scientific research, and give them a relaxed environment for innovation [63] so as to continuously improve their creative thinking and creative ability in scientific research. In addition, colleges and universities should pay attention to the cultivation of the feedback ability of academic supervisors. Through training in developmental feedback teaching methods, communication skills, and other related areas, the operational level and application ability of academic supervisors in teaching practice is constantly improved.

Second, the mediating role of intrinsic motivation provides colleges and universities with a new perspective for reflection, making them realize that ASDF can stimulate the intrinsic motivation of postgraduate students to participate in creative activities, and subsequently show a higher level of creativity. From this point of view, this study suggests that the intrinsic motivation of postgraduate students should be highly valued and constantly stimulated in practice. In daily student management, it is necessary to fully respect the subjective status of postgraduate students in creative activities, explore their inner creative interests, and promote the transformation of postgraduates from passive to active participants in creativity. Academic supervisors should play an incentivizing role and give positive developmental feedback in a timely and clear manner, especially when postgraduates show initiative and creativity in scientific research. In addition, academic supervisors should give postgraduate students full autonomy in practice, tolerate their mistakes and shortcomings in the creative process, stimulate their intrinsic motivation to actively engage in creative activities, and finally enhance their creativity.

Finally, the moderating role of creative self-efficacy means that it is of great significance in the improvement of the creative self-efficacy of postgraduate students through multiple means. To begin with, in daily teaching and management practice, the postgraduate management department should provide regular updates on the creative–efficacy dynamics of postgraduate students through various channels, such as one-to-one discussions, work reports, and questionnaire surveys. Additionally, academic supervisors should make full use of opportunities to communicate with postgraduate students and give timely affirmation and recognition to their creative achievements. It is also necessary to observe and explore the strengths of postgraduate students, which may not only help them find their own advantages and stimulate their creative confidence, but also help them correctly understand the setbacks and failures in the process of creation and strive to shape their overall positive creative self-efficacy. In addition, colleges and universities should actively organize postgraduate students to carry out creative exchange activities and encourage postgraduate students to share their experiences, as this can enhance the construction of the value and significance of creative work. In the process of helping others create, postgraduate students realize their own value and improve their creative self-efficacy.

### 5.3. Limitations and Recommendations

Despite the above theoretical and managerial contributions, this study still has several limitations. First, the sample of this study was collected from only one research university in Beijing, China. Many previous studies have confirmed that the uniqueness of traditional Chinese culture [8,36], especially the continued respect for authority, collectivism, and harmony, may affect how Chinese people [64], including postgraduate students, perceive and react to feedback from their academic supervisors [13,25,37]. Therefore, this study suggests that future studies use samples from different cultural backgrounds to re-test the proposed theoretical model and discuss its cross-cultural applicability.

Second, this study adopted a time-lagged research design, which to some extent overcomes the limitations of cross-sectional research design in the field of education research. However, this study still fails to reveal the causal relationship between ASDF, intrinsic motivation, and PSC. For instance, individuals with previous success in creativity might reinforce their intrinsic motivation to actively seek change [36,65,66], which in turn may trigger their perception of developmental feedback. Since the whole proposed hypothetical model is theoretically based, it is certain that the above issues cannot significantly affect the interpretation of this study. However, this study still encourages future scholars to adopt a longitudinal or experimental design to explore the causal relationships among the core variables in detail.

Finally, this study only built and validated a moderated mediation model to discuss the moderating effect of creative self-efficacy and the mediating effect of intrinsic motivation in the relationship between ASDF and PSC. However, a large number of early leadership studies in higher education have confirmed that the influence of academic supervisor behavior or leadership style on PSC is a complex process in which other mediators or moderators may exist. For example, Meng and Zhao verified the mediating roles of professional knowledge, intrinsic motivation, and creative thinking in the influence of academic supervisors’ empowering, considering, motivating, and developing behavior on PSC [8]. Shang et al. confirmed the mediating role of promotion-focused behavior and the moderating role of power sources in the relationship between authentic leadership and PSC [9]. This study therefore recommends that future research investigate other mediators or moderators in the ASDF–PSC relationship.

## 6. Conclusions

In conclusion, in the context of a Chinese university, this study built and confirmed a moderated mediation model to explore the influence of academic supervisor developmental feedback on postgraduate student creativity. The mediating effect of intrinsic motivation and the moderating effect of creative self-efficacy in such influence processes were also discussed in detail. The empirical results indicated that ASDF has a positive influence on the intrinsic motivation of postgraduate students and on PSC. Intrinsic motivation not only exerts a positive effect on PSC, but also mediates the influence of ASDF on PSC. Creative self-efficacy positively moderates the effect of ASDF on intrinsic motivation and also affects PSC, which means that for postgraduate students with high creative self-efficacy, ASDF stimulates their intrinsic motivation more obviously, and their creativity is higher than those with low creative self-efficacy. These findings highlight the importance of developmental feedback from academic supervisors in improving the intrinsic motivation and creativity of postgraduate students in Chinese universities and enriches the literature on feedback, motivation, and creativity in the field of education.

## Figures and Tables

**Table 1:** Results of confirmatory factor analyses.

Model	Factor	χ^2^/*df*	CFI	GFI	RMSEA	SRMR
Four-factor model	ASDF, CSE, IM, PSC	1.067	0.998	0.972	0.013	0.044
Three-factor model	ASDF, CSE+IM, PSC	4.159	0.884	0.868	0.092	0.088
Two-factor model	ASDF+CSE+IM, PSC	5.880	0.824	0.824	0.114	0.113
Single-factor model	ASDF+CSE+IM+PSC	11.987	0.599	0.677	0.172	0.188

Note: *N* = 374; CSE = creative self-efficacy; IM = intrinsic motivation; ideal model-fit indicators are: χ^2^/*df* < 3, CFI > 0.9, TLI > 0.9, RMSEA < 0.08, SRMR < 0.08.

**Table 2:** Descriptive statistics and correlation coefficients of variables.

Title 1	Mean	*SD*	1	2	3	4	5	6
1. Gender	0.55	0.498	1.000					
2. Grade	1.95	0.792	0.050	1.000				
3. Major	3.68	1.643	−0.009	0.204 **	1.000			
4. ASDF	2.51	0.898	0.060	0.538 **	0.239 **	1.000		
5. CSE	2.57	0.967	0.132 *	0.221 **	0.113 *	0.409 **	1.000	
6. IM	2.48	1.084	0.037	0.242 **	0.218 **	0.390 **	0.495 **	1.000
7. PSC	3.08	1.034	0.278 **	0.304 **	0.162 ***	0.337 **	0.325 **	0.289 **

Note: *N* = 374; *SD* = standard deviation; * *p* < 0.05, ** *p* < 0.01, *** *p* < 0.001.

**Table 3:** Results of hierarchical regression analyses.

Variables	Intrinsic Motivation			PSC				
	Model 1	Model 2	Model 3	Model 4	Model 5	Model 6	Model 7	Model 8
Gender	0.028	0.016	−0.048	0.266 ***	0.260 ***	0.266 ***	0.258 ***	0.255 ***
Grade	0.205 ***	0.032	0.036	0.268 ***	0.226 ***	0.268 ***	0.158 **	0.152 **
Major	0.176 **	0.120 **	0.125 **	0.110 *	0.073	0.110 *	0.080	0.058
ASDF		0.340 ***	0.137 *				0.218 ***	0.161 **
IM					0.208 ***			0.167 **
CSE			0.381 ***					
ASDF × CSE			0.163 ***					
R^2^	0.089	0.169	0.327	0.173	0.213	0.173	0.206	0.229
ΔR^2^		0.080	0.157		0.040		0.033	0.023
F	12.08 ***	18.82 ***	29.69 ***	25.87 ***	24.95 ***	25.97 ***	23.97 ***	21.89 ***

Note: *N* = 374; *SD* = Standard Deviation; * *p* < 0.05, ** *p* < 0.01, *** *p* < 0.001.

**Table 4:** Results of bootstrap analyses.

Conditional Indirect Effect	Effect	SE	95%	
			LLCI	ULCI
*M* – 1 SD	0.002	0.014	−0.027	0.313
Mean	0.026	0.014	0.005	0.063
*M* + 1 SD	0.051	0.021	0.018	0.106
**Index of moderated mediation**				
Creative self-efficacy	0.025	0.019	0.007	0.054

Note: *N* = 374; bootstrap sample size = 5000; LL = lower limit; CI = confidence interval; UL = upper limit.

## Data Availability

Data supporting reported results are available from the authors on request.
